# Global Sensitivity Analysis for the Polymeric Microcapsules in Self-Healing Cementitious Composites

**DOI:** 10.3390/polym12122990

**Published:** 2020-12-15

**Authors:** Shuai Zhou, Yue Jia, Chong Wang

**Affiliations:** College of Materials Science and Engineering, Chongqing University, Chongqing 400045, China; 20162802@cqu.edu.cn

**Keywords:** polymeric healing agents, self-healing composites, global sensitivity analysis, extended Fourier amplitude sensitivity test, micromechanics

## Abstract

Cementitious composites with microencapsulated healing agents are appealing due to the advantages of self-healing. The polymeric shell and polymeric healing agents in microcapsules have been proven effective in self-healing, while these microcapsules decrease the effective elastic properties of cementitious composites before self-healing happens. The reduction of effective elastic properties can be evaluated by micromechanics. The substantial complicacy included in micromechanical models leads to the need of specifying a large number of parameters and inputs. Meanwhile, there are nonlinearities in input–output relationships. Hence, it is a prerequisite to know the sensitivity of the models. A micromechanical model which can evaluate the effective properties of the microcapsule-contained cementitious material is proposed. Subsequently, a quantitative global sensitivity analysis technique, the Extended Fourier Amplitude Sensitivity Test (EFAST), is applied to identify which parameters are required for knowledge improvement to achieve the desired level of confidence in the results. Sensitivity indices for first-order effects are computed. Results show the volume fraction of microcapsules is the most important factor which influences the effective properties of self-healing cementitious composites before self-healing. The influence of interfacial properties cannot be neglected. The research sheds new light on the influence of parameters on microcapsule-contained self-healing composites.

## 1. Introduction

**Figure 1:**
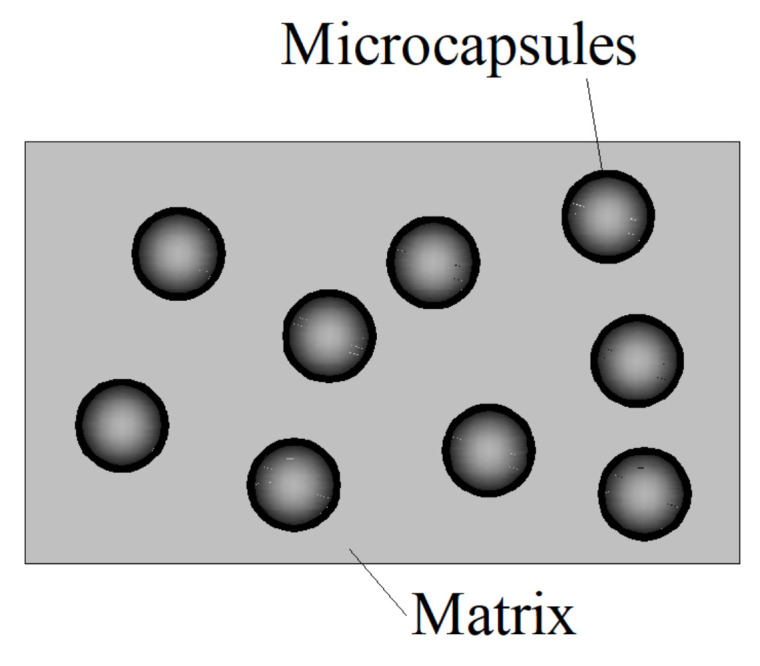
Schematic illustration of microcapsule-contained cementitious composites.

Self-healing is the remarkable ability of living organisms to repair their own damage by themselves. Inspired from nature, the great challenge now is to create and develop composites with high potential for self-healing, offering alternatives to the current options and moving toward materials with extended service lifetime for various applications. Most of these are innovative materials with excellent healing performance, such as ionomers, semiconductors, self-assembling systems, hydrogels, micro- and nanoparticles, coatings, films and membranes, microcapsules, vascular networks, shape memory or stimuli-induced self-healing materials [1,2,3,4,5]. Concretes with microencapsulated healing agents are appealing since they are able to spontaneously repair themselves after damage or degradation, recovering structural integrity and functionality by increasing the rate of healing versus the rate of damage [6,7,8,9,10,11,12]. The microcapsule-contained self-healing cementitious composite involves the polymeric healing agent (repairing the cracks), the catalyst (speeding up the polymerization) and the polymeric shell (encapsulating the healing agent), as exhibited in Figure 1. The healing agents can be cyanoacrylates, epoxy, methyl methacrylate and multicompound healing agents, while polyurethane, polyurea resin and formaldehyde resin can be adopted as shells to produce the self-healing microcapsules [13].

Even though microcapsule-contained cementitious composites can achieve self-healing, the elastic properties of the cementitious composite before self-healing happens are influenced because of these soft inclusions based on our previous research [14,15,16,17,18,19,20,21,22,23,24]. The effective elastic properties can be evaluated by micromechanics. There are many theoretical methods to tackle the effective elastic moduli of multiphase composites, such as mathematical lower and upper bounds, the self-consistent method, the differential scheme, the Mori–Tanaka method and the generalized self-consistent method, etc. A new micromechanical model which can reflect the properties of pothole patching material is proposed [25]. However, it does not apply to microcapsule-contained composites directly since it ignores the microstructure of microcapsules. Existing experimental results show that interfaces exist between the microcapsules and the concrete matrix, which significantly affect the mechanical properties of the microcapsule-contained self-healing cementitious composite at the micro and macro level [26]. Hence, the interfaces should be considered to calculate the effective properties of microcapsule-contained cementitious materials. Recently, the effect of interfacial damage on the effective elastic modulus of spherical-particle-reinforced composites has been investigated based on the linear-spring model by Yanase and Ju [27]. Further, many other theoretical models concentrate on the interface between inclusions and the matrix [28,29,30,31,32,33]. The microcapsules have a multilayer microstructure [34,35]. However, a micromechanical model which can consider both the interfacial performance and the structure of microcapsules has not been built.

The substantial complicacy often included in micromechanical models leads to the need for specifying a large number of parameters and inputs. Additionally, there are nonlinearities in input–output relationships. Hence, it is a prerequisite to know the sensitivity of these models. Sensitivity analysis (SA) is a fundamental tool in the building, use and understanding of models of all forms. It provides information about the behavior of the model being evaluated, such as the identification of relevant model inputs, information on the model balance [36], indications for model simplification, model building and identification parts of the model which could be improved. There are two groups of sensitivity analyses: the local sensitivity analysis and the global sensitivity analysis. The local SA checks the local response of the outputs by varying input parameters one at a time and holding other parameters at central values. Hence, the sensitivity index is dependent on the central values chosen by other parameters. The global SA examines the global response of model outputs by exploring a finite region. Hence, the global SA is more precise than the local SA. There are many global SA methods, such as the Morris method, sampling-based methods and variance-based methods, etc. The Fourier Amplitude Sensitivity Test (FAST) and Extended Fourier Amplitude Sensitivity Test (EFAST) belong to the variance-based method. Sensitivity analysis has been applied to many models [36,37,38,39]. The Partial Rank Correlation Coefficient (PRCC) method appears to be the most efficient and reliable among the sampling-based indices [40]. Correlation provides a measure of the strength of a linear association between an input and an output. PRCC is a sampling-based method for nonlinear relationships between an input and an output. It is adopted to validate the results of the EFAST in this paper. Our predictions of the micromechanical model will be strengthened if we can reduce uncertainty and get better estimates on specific parameters of the model.

In this research, a new micromechanical model of the microcapsule-contained self-healing cementitious composite was developed. The sensitivity analysis of the model was conducted using EFAST. The influence of microcapsules before self-healing happens was focused on. An outline of this paper is as follows. In Section 2, the micromechanical model of the microcapsule-contained cementitious composite is proposed, and the influence of the interface can be considered. In Section 3, EFAST is introduced. Moreover, relative parameters of the micromechanical model are presented. In Section 4, the results and discussion are illustrated. Conclusions of the study are drawn in Section 5.

## 2. A Micromechanical Model for the Microcapsule-Contained Cementitious Composite

### 2.1. Multilevel Homogenization Scheme for Predicting the Effective Properties

**Figure 2:**
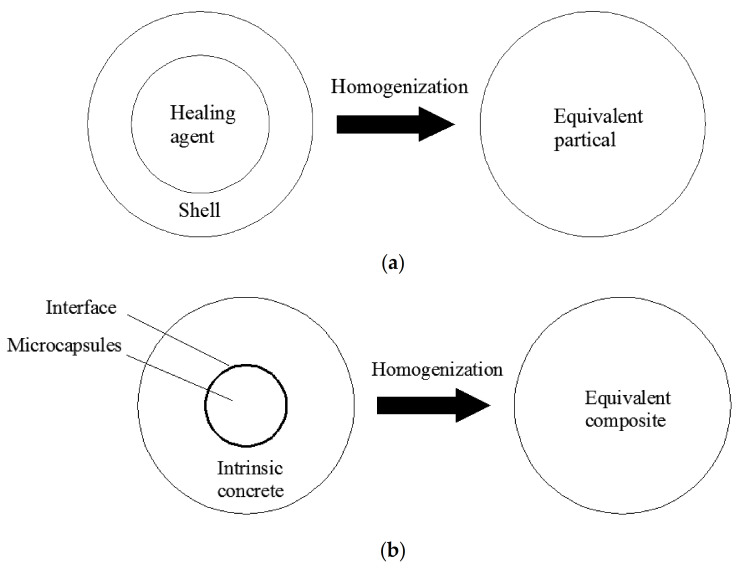
The homogenization process: (**a**) the first level: the homogenization of the shell and healing agents inside, and (**b**) the second level: the homogenization of the intrinsic concrete, the equivalent inclusion and interfaces.

To obtain the effective properties of the microcapsule-contained cementitious composite, the multilevel homogenization method was applied [26]. The two-level homogenization process can be summarized as follows: (1) First, the effective properties of microcapsules, composed of shells and healing agents inside, were obtained by the homogenization of the two-phase composite as illustrated in Figure 2a; (2) second, the effective properties of microcapsule-contained cementitious materials can be estimated by homogenizing the three-phase composite composed of the intrinsic concrete, the interface and the equivalent inclusion, as demonstrated in Figure 2b.

### 2.2. The First-Level Homogenization

The microcapsules consisted of two parts: the polymeric healing agents inside and the polymeric shell outside. To calculate the effective properties of a coated particle, an analytical solution was derived. The process was similar with that use in previous research [25]. A 2D cylindrical shell was considered under the internal pressure *p* and the external pressure *q*. The displacement field, *u_r_*, can be expressed as [41]

(1) $$u_{r}=\frac{\left(1-\upsilon \right)}{E}\frac{(pa^{2}-qb^{2})r}{b^{2}-a^{2}}+\frac{\left(1+\upsilon \right)}{E}\frac{a^{2}b^{2}(p-q)}{(b^{2}-a^{2})r},$$

where *E*, *ν*, *a* and *b* are Young’s modulus, Poisson’s ratio, the radius of the inner ring and the radius of the outer ring, respectively. *p* and *q* are the internal and external pressures on the ring. The core–shell ratio is defined as *k* = *a*/*b*.

**Figure 3:**
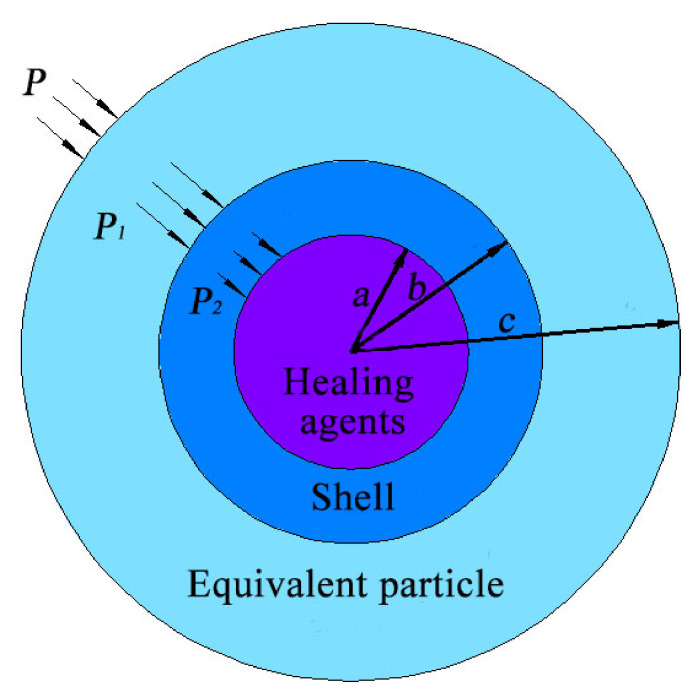
A multilayer-coated model for self-healing microcapsules.

Then, another outside layer, called the equivalent coated particle, is necessary to obtain the effective properties of the coated particle. The elastic properties are equal to the effective properties of the inner microcapsule, as shown in Figure 3.

According to the displacement continuity conditions on each interface given in Figure 3 and Equation (1), the displacement on each layer can be calculated:

(2) $$-\frac{\left(1-\upsilon_{2}\right)}{E_{2}}P_{2}a=\frac{1}{E_{1}}\left[(1-\upsilon_{1})\frac{\left(P_{2}a^{2}-P_{1}b^{2}\right)a}{b^{2}-a^{2}}+\left(1+\upsilon_{1}\right)\frac{a^{2}b^{2}\left(P_{2}-P_{1}\right)}{(b^{2}-a^{2})a}\right],at\;r=a$$

(3) $$\frac{1}{E_{1}}\left[(1-\upsilon_{1})\frac{\left(P_{2}a^{2}-P_{1}b^{2}\right)b}{b^{2}-a^{2}}+\left(1+\upsilon_{1}\right)\frac{a^{2}b^{2}\left(P_{2}-P_{1}\right)}{(b^{2}-a^{2})b}\right]=\frac{1}{E_{eq}}\left[(1-\upsilon_{eq})\frac{\left(P_{1}b^{2}-Pc^{2}\right)b}{c^{2}-b^{2}}+\left(1+\upsilon_{eq}\right)\frac{b^{2}c^{2}\left(P_{1}-P\right)}{(c^{2}-b^{2})b}\right],at\;r=b$$

(4) $$-\frac{\left(1-\upsilon_{eq}\right)}{E_{eq}}Pc=\frac{1}{E_{eq}}\left[(1-\upsilon_{eq})\frac{\left(P_{1}b^{2}-Pc^{2}\right)c}{c^{2}-b^{2}}+(1+\upsilon_{eq})\frac{b^{2}c^{2}\left(P_{1}-P\right)}{(c^{2}-b^{2})c}\right],at\;r=c$$

where *E_eq_*, *E*_1_ and *E*_2_ denote Young’s moduli of the equivalent particle, the shell and the healing agent, respectively. Further, *v_eq_*, *v*_1_ and *v*_2_ mean the Poisson’s ratios of the equivalent particle, the shell and the healing agent, respectively.

By solving above equations, Young’s modulus of the equivalent particle can be expressed as

(5) $$E_{eq}=\frac{E_{1}(1-a^{2}/b^{2})(1-\upsilon_{eq})}{(a^{2}/b^{2})(1+\upsilon_{1})+(1-\upsilon_{1})-\frac{4E_{2}a^{2}/b^{2}}{E_{1}(1-a^{2}/b^{2})(1-\upsilon_{2})+E_{2}\left[\left(1+\upsilon_{1})+a^{2}/b^{2}(1-\upsilon_{1}\right)\right]}}\;\;\;\;\;\;\;\;\;\;\;\;\;\;\;\;\;\;\;\;\;\;\;\;\;\;\;\;\;\;\;\;$$

Meanwhile, *ν_eq_* can be obtained by [42]

(6) $$v_{eq}=\frac{v_{1}f_{1}E_{1}+v_{2}f_{2}E_{2}}{f_{1}E_{1}+f_{2}E_{2}}$$

where *f*_1_ and *f*_2_ are the volume fraction of the shell and healing agents, respectively.

### 2.3. The Second-Level Homogenization

**Figure 4:**
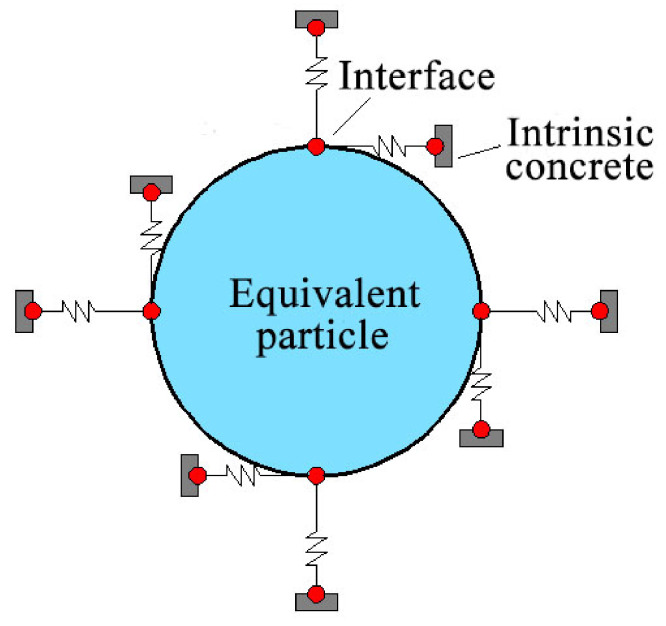
A schematic illustration of the interface model.

Then, the effective properties of cementitious materials are considered. Three traditional solid phases, i.e., mortar, coarse aggregates and their interfaces, are merged into one matrix phase, namely the intrinsic concrete, in the representative volume element [26]. To simplify the model, we consider the effective properties of the intrinsic concrete directly. The interface between the microcapsules and the intrinsic concrete is involved here, as demonstrated in Figure 4.

The effective elastic stiffness tensor of a composite can be obtained [27]

(7) $$C^{*}=\left[\sum_{r=0}^{N}\phi^{\left(r\right)}C^{\left(r\right)}•T_{MT}^{\left(r\right)}\right]•{\left\{\phi^{\left(0\right)}I+\sum_{r=1}^{N}\phi^{\left(r\right)}\left[{\left(C^{\left(r\right)}\right)}^{-1}+R^{\left(r\right)}\right]•C^{\left(r\right)}•T_{MT}^{\left(r\right)}\right\}}^{-1}\;\;\;\;\;\;\;\;\;\;$$

where 𝝓^(*r*)^ and **C**^(*r*)^ are the volume fraction and the elastic stiffness tensor of *r-*th phase, respectively. 0-th represents the intrinsic concrete. **I** denotes the fourth-order identity tensor. $T_{MT}^{\left(r\right)}$ and $R^{\left(r\right)}$ are two tensors relative to interfacial properties, and can be calculated by [27]

(8) $$T_{MT}^{\left(0\right)}=I$$

(9) $$T_{MT}^{\left(1\right)}=I-S^{MD}•{\left[S^{MD}+{\left(C^{\left(1\right)}-C_{0}\right)}^{-1}•C_{0}\right]}^{-1}$$

(10) $$R_{ijkl}=\frac{1}{a}\left[\frac{\beta -\alpha }{5}\delta_{ij}\delta_{kl}+\frac{3\alpha +2\beta }{10}\left(\delta_{il}\delta_{jk}+\delta_{ik}\delta_{jl}\right)\right]$$

where **S***^MD^* is the modified Eshelby tensor and can be obtained by the Direct Computation method [27]

(11) $$S_{ijkl}^{MD}=\hat{\lambda }\delta_{ij}\delta_{kl}+\hat{\mu }\left(\delta_{il}\delta_{jk}+\delta_{ik}\delta_{jl}\right)$$

(12) $$\hat{\lambda }=\frac{\left(5\left(5\upsilon_{0}-1\right)\left(-3+9\upsilon_{0}-6\upsilon_{0}^{2}\right)+8\alpha^{\prime }\mu_{0}\left(1-2\upsilon_{0}^{2}\right)\left(5\upsilon_{0}-4\right)+2\beta^{\prime }\mu_{0}\left(1+71\upsilon_{0}-101\upsilon_{0}^{2}+45\upsilon_{0}^{3}\right)\right)}{\left[-3+9\upsilon_{0}-6\upsilon_{0}^{2}+2\beta^{\prime }\mu_{0}{\left(1+\upsilon_{0}\right)}^{2}\right]\left[75\left(1-\upsilon_{0}\right)+4\mu_{0}\left(5\upsilon_{0}-4\right)\left(3\alpha^{\prime }+2\beta^{\prime }\right)\right]}$$

(13) $$\hat{\mu }=\frac{\left(5-6\alpha^{\prime }\mu_{0}-4\beta^{\prime }\mu_{0}\right)\left(4-5\upsilon_{0}\right)}{75\left(1-\upsilon_{0}\right)+4\mu_{0}\left(5\upsilon_{0}-4\right)\left(3\alpha^{\prime }+2\beta^{\prime }\right)}$$

(14) $$\alpha^{\prime }=\frac{\alpha }{b},\;\;\beta^{\prime }=\frac{\beta }{b}$$

where *ɑ* and *β* are related to the interfacial sliding and the interfacial separation, respectively. *b* represents the radius of microcapsules.

By adopting the above micromechanical model, the effective properties of the microcapsule-contained cementitious composites can be obtained.

## 3. Global Sensitivity Analysis Method

FAST is one of the most elegant methods for the sensitivity analysis [43]. It can be applied to many nonlinear models. EFAST inherits the advantages of FAST with some modifications. It quantifies the contribution of each input parameter to the total variances of the output by variance-based methods. In this paper, EFAST is applied to carry out the sensitivity analysis. The main idea of EFAST is to explore the multidimensional space by a suitably defined search-curve. If the *i*-th factor has a strong influence on the output, the amplitude of oscillation of *y* = *f*(*x*) at frequency *w_i_* is great. The main process of EFAST is summarized as follows:

In a model with *n* inputs, *y* = *f*(*x*_1_, *x*_2_,…, *x*_*n*_), with parameters in the domain of unit hypercube

(15) $$K^{n}=(\left.x\right|0\leq x_{i}\leq 1;i=1,\ldots ,n)$$

Then, a new function is introduced

(16) $$x_{i}=G_{i}(s),\;i=1,2,\ldots ,n$$

*G_i_* is a search-curve. There are many forms of *x_i_*. Here, we take the transformation proposed by Saltelli et al. [43]

(17) $$x_{i}=\frac{1}{2}+\frac{1}{\pi }\arcsin(sin\;w_{i}s)$$

where *w*_i_ is a set of different, linearly independent of integer frequencies associated with each factor *x*_i_. *s* varies in (−*π*/2, *π*/2). By using Fourier transform, the first-order sensitivity index ${\hat{S}}_{i}$ can be obtained [43]

(18) $${\hat{S}}_{i}={\hat{D}}_{i}/\hat{D}$$

where $\hat{D}$ and ${\hat{D}}_{i}$ are the total variance and the variance caused by the *i*-th parameter, respectively. The detailed process can be found in previous research [43].

The total variance can be decomposed into the variance caused by a single parameter and combined parameters. Then, the higher-order sensitivity index can be calculated [44]. In this paper, the direct influence of inputs is studied, while the higher-order influence is ignored.

The model outputs—the bulk modulus *K* and the shear modulus *G*—were considered in this sensitivity analysis. They were chosen as they are the main parameters to evaluate the elastic properties of a material. There are only two independent elastic constants in isotropic media, such as ordinary concretes and polymers. By using *G* and *K*, other elastic parameters can be obtained. Further, the elastic performance of materials can be simulated [23]. Some rational parameters are presented to illustrate the process in Table 1. The elastic properties of some common concretes are listed in Table 2.

## 4. Results and Discussion

Here, the normalized EFAST first-order effects with respect to the input parameters were investigated. The normalization was conducted by [39]

(19) $$S_{n,i}=S_{i}/\sum_{i=1}^{6}S_{i}$$

**Figure 5:**
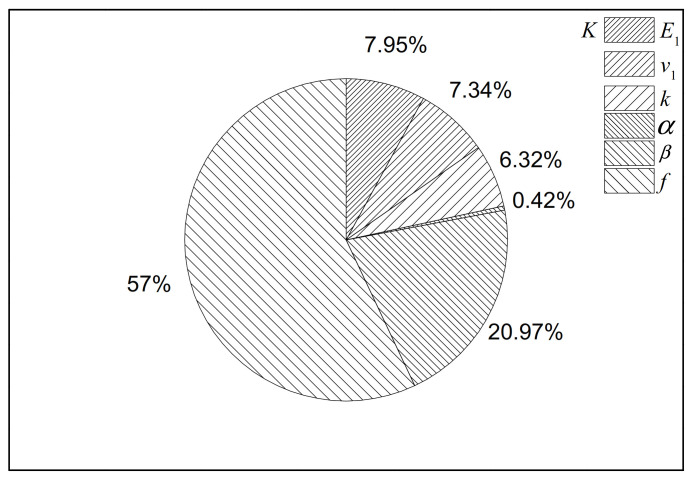
First-order sensitivity indices (FSIs) computed by the Extended Fourier Amplitude Sensitivity Test (EFAST) for the bulk modulus in the C30 concrete.

The first-order sensitivity indices (FSIs) of the bulk modulus in the C30 concrete were obtained by applying the EFAST variance-based sensitivity analysis in Figure 5.

By analyzing the results in Figure 5, the influence of input parameters is known. The volume fraction of microcapsules (*f*) and the interfacial separation property (*β*) had the greatest effect (around 57% and 21%, respectively) on the bulk modulus of the C30 concrete. Hence, the volume fraction of microcapsules should be selected carefully to maintain the desired bulk modulus. The interfacial separation property (*β*) is also a sensitive factor for the microcapsule-contained cementitious materials. The interfacial bonding should be good to avoid weak elastic behaviors. The properties of the shell (i.e., *E*_1_, *v*_1_ and *k*) have a medium influence on the outputs. They make up approximately 20% altogether. The interfacial sliding property *ɑ* only takes up about 0.42% of the influence, which can be neglected. The result was acceptable since the interfacial sliding parameter has little influence on the bulk modulus as illustrated in previous studies [27].

**Figure 6:**
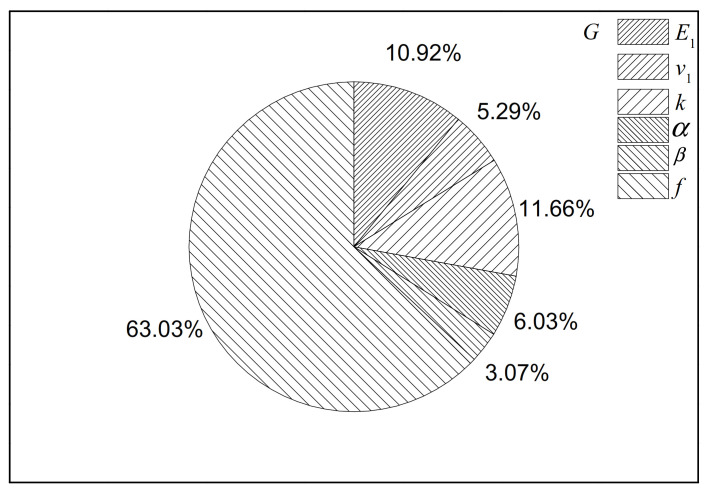
FSIs computed by the EFAST sensitivity analysis for the shear modulus in the C30 concrete.

The FSIs of inputs for the shear modulus of the C30 concrete are evaluated in Figure 6. According to Figure 6, the FSI of the volume fraction of microcapsules (*f*) is the largest (around 63%). Hence, the volume fraction should be given extra attention in order to obtain the desired effective shear properties. The elastic modulus and the core-shell ratio of microcapsules also have a medium influence on the effective properties (approximately 11%). The interfacial sliding property *ɑ* has a greater impact on the shear modulus (5%) than that on the bulk modulus (0.42%). The results prove that elastic properties may have insensitive parameters in some objective functions and not in others, as illustrated in Figure 5 and Figure 6. It is clear that the two objective functions are sensitive to the volume fraction of microcapsules.

**Figure 7:**
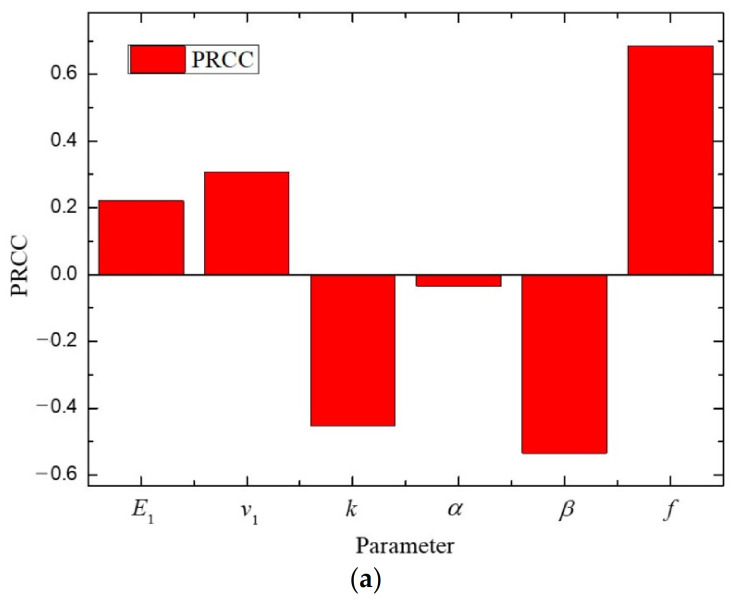

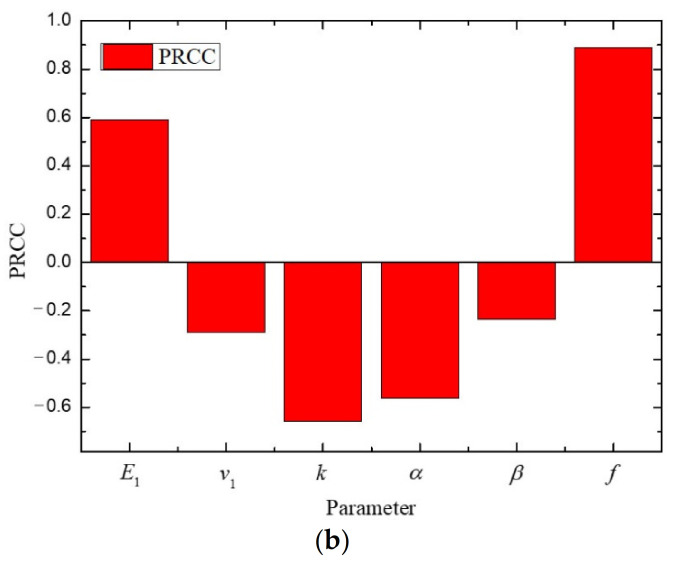
The Partial Rank Correlation Coefficient (PRCC) sensitivity analysis for the (**a**) bulk modulus and (**b**) shear modulus in the C30 concrete.

To validate the results, PRCC was applied in this research. Figure 7 shows the overall sensitivity of each parameter of the bulk modulus and the shear modulus in the C30 concrete. The result of PRCC was similar to that obtained by EFAST (see FSI values in Figure 5 and Figure 6). However, there was a slight difference in the ordering of the parameter sensitivities. The order of sensitivity for the bulk modulus by EFAST method was *E*_1_ > *v*_1_ > *k*, while PRCC values yielded a slightly different order *k* > *v*_1_ > *E*_1_. The other rankings were the same. These results support that the results of EFAST are correct, and PRCC provides a similar identification of sensitive parameters.

**Figure 8:**
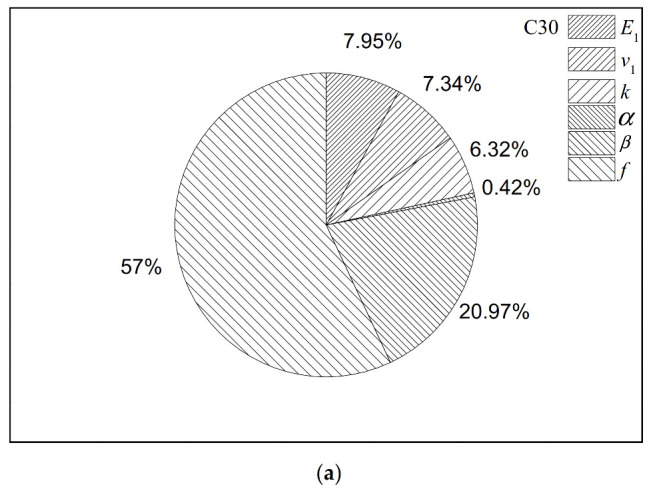

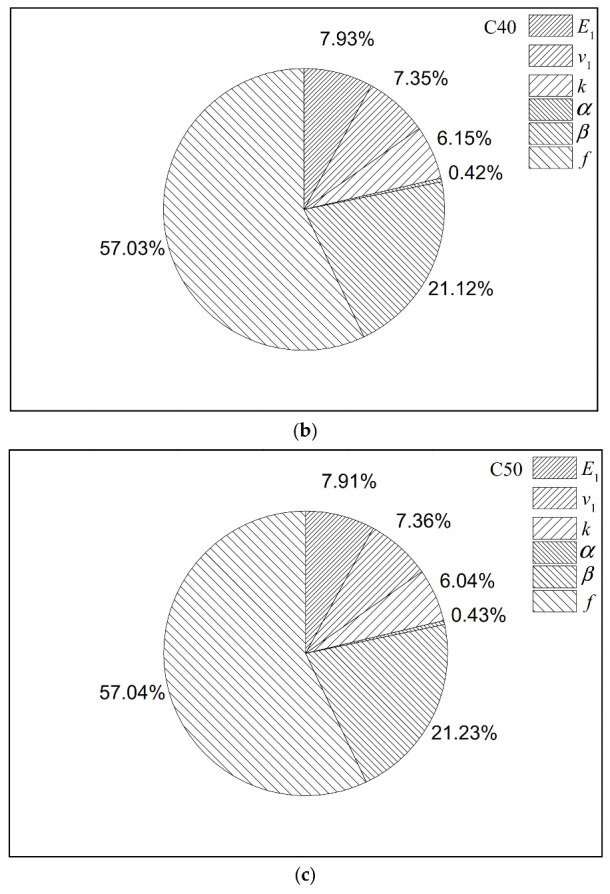
FSIs computed by the EFAST sensitivity analysis for the bulk modulus of the (**a**) C30 concrete, (**b**) the C40 concrete, and (**c**) the C50 concrete.

The sensitivity of parameters of the micromechanical model may change between different cementitious matrices. However, the difference is not obvious and can be neglected. Let us examine this finding in the case of the bulk modulus. In all three situations in Figure 8 (i.e., the C30 concrete, the C40 concrete and the C50 concrete), the FSI of each parameter is similar. This implies that even though the FSI of each parameter may be varied in different concretes, the general rankings can be summarized. The volume fraction and the interfacial separation property should be paid extra attention to. Considering the properties of concretes, the order of FSIs of parameters is the same in different concretes.

**Figure 9:**
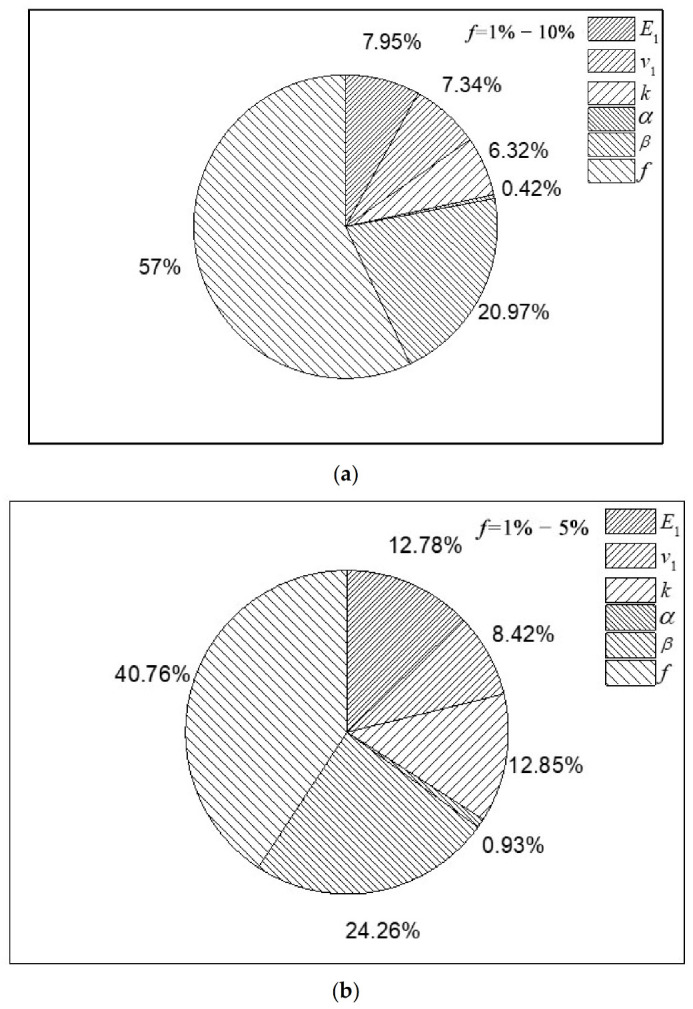
FSI values using an (**a**) original and (**b**) adjusted range of the volume fraction of microcapsules for the C30 concretes with the bulk modulus objective function.

Global sensitivity analysis such as EFAST requires the range of parameter values to be explored. Changing the parameter range may affect the sensitivity index [45]. We investigated the influence of the parameter range of our micromechanical model by repeating the sensitivity analysis with other parameter ranges. The objective function of bulk modulus used for sensitivity analysis was taken to illustrate the influence of the range in the C30 concrete. Here, the range of the volume fraction changed from (1%, 10%) to (1%, 5%). The results are displayed in Figure 9. For the bulk modulus, if the volume-fraction parameter range changes from (1%, 10%) to (1%, 5%), the FSI of the volume fraction decreases by 16.24%. Meanwhile, the elastic modulus, the core–shell ratio and the interfacial separation property become more sensitive. Their FSI values increase up to 4.83%, 6.53% and 3.29%, respectively. Therefore, the analysis confirms that FSI values of parameters can change substantially if the range of the volume fraction of microcapsules changes. However, the volume fraction still has the greatest impact on the elastic properties.

## 5. Conclusions

The study presents the results of sensitivity analysis using EFAST on the proposed micromechanical model of microcapsule-contained self-healing cementitious composites. The proposed micromechanical model can consider the microstructures and interfaces of microcapsules. EFAST helps to identify which parameters are required for knowledge improvement to achieve the desired level of confidence in the results. The results show that the volume fraction of microcapsules is the most important factor which influences the effective properties of the microcapsule-contained self-healing cementitious composites. The influence of interfacial properties cannot be neglected. The sensitivity analysis is affected by the parameter ranges. Not all elastic properties are sensitive to the same inputs. Even though the properties of the matrix make a difference, the general rankings of FSIs of parameters are the same among different concretes. From the research, the volume fraction of microcapsules should be chosen after a precise design. Meanwhile, the surface treatment should be conducted on the self-healing microcapsules to enhance the interfacial bonding between the microcapsules and the cementitious matrix. The optimal volume fraction of microcapsules is related to the healing probability, the healing ratio, properties of healing agents, the radius of microcapsules, the size of cracks in the matrix, etc. This investigation mainly concentrated on the influence of different parameters on the elastic properties before self-healing. The optimum volume fraction can be obtained using our previous models [20,21,22,23,24]. In the future, a viscoelastic model will be developed based on the present research to extend its application.

## Figures and Tables

**Table 1:** Parameters used for the sensitivity analysis.

Parameter	Description	Unit	Scope
*E*_1_	Elastic modulus of the shell	GPa	(1, 10)
*v*_1_	Poisson’s ratio of the shell	-	(0.001, 0.499)
*k*	The core-shell ratio	-	(0.1, 0.9)
*ɑ*	The interfacial sliding compliance	1/MPa	(0.001, 0.01)
*β*	The interfacial separation compliance	1/MPa	(0.001, 0.01)
*f*	The volume fraction of microcapsules	-	(1%, 10%)

**Table 2:** Elastic properties of some common concretes.

Type	Elastic Modulus	Poisson’s Ratio
C30	30 GPa	0.2
C40	32.5 GPa	0.2
C50	34.5 GPa	0.2
